# *Acacia dealbata* invasion in Chile: Surprises from climatic niche and species distribution models

**DOI:** 10.1002/ece3.5295

**Published:** 2019-06-23

**Authors:** Bárbara Langdon, Aníbal Pauchard, Ramiro O. Bustamante

**Affiliations:** ^1^ Laboratorio de Ecología Geográfica, Facultad de Ciencias Universidad de Chile Santiago Chile; ^2^ Laboratorio de Invasiones Biológicas (LIB), Facultad de Ciencias Forestales Universidad de Concepción Concepción Chile; ^3^ Instituto de Ecología y Biodiversidad (IEB) Santiago Chile

**Keywords:** *Acacia dealbata* invasion, invasion management strategies, niche modeling, potential distribution models, south‐central Chile

## Abstract

**Aim:**

Tree invasions are a threat to biodiversity conservation, and although it is hard to predict the future spread of invasive tree species, there are tools available which could allow some estimations. The magnitude of spatial spread (a proxy of invasiveness) can be predicted from species climatic requirement (climatic niche) and can be represented by species distribution models (SDMs). We aimed to assess whether *Acacia dealbata* conserves its niche in the new environment of south‐central Chile, and also, to estimate the invasive stage of the species.

**Location:**

South‐central area of Chile, between the O'Higgins (34°0″0′S) and Aysen Regions (47°0″0′S).

**Methods:**

We used a combination of global, native, and regional data to improve the estimation of the potential distribution of *A. dealbata*, which has been considered one of the most invasive species of the genus, being registered in at least 34 countries in all the Continents.

**Results:**

Our results show that *A. dealbata* does not conserve its niche in the study area, invading areas with climatic conditions different from those of the native range. It is also not at equilibrium with the environment. According to the global versus regional SDM comparisons, populations present in south‐central Chile present different invasion stages. There are some stable populations, but there are other populations colonizing new areas, occupying unsuitable habitats and some of them are adapting to new climatic conditions. Climatic factors, such as precipitation seasonality, could be acting behind the expansion to new environments, and biotic factors or dispersal limitations could be preventing the species to colonize suitable areas.

**Main conclusions:**

The invasion process of *A. dealbata* is far from stabilizing, and management options should focus on prevention, avoiding, for example, the introduction of the species to Patagonia where the species has not spread yet. More research is needed to complement our results and enhance the development of effective management strategies.

## 1 INTRODUCTION

Tree invasions represent a major threat to biodiversity conservation (Dickie et al., 2014; Rejmanek & Richardson, 2013; Richardson & Rejmanek, 2011; Rundel, Dickie, & Richardson, 2014). Despite the short time since the invasion process of many trees began (Rejmanek & Richardson, 2013; Richardson, Hui, Nuñez, & Pauchard, 2014), there is a large number of them already listed as aggressive species (Richardson & Rejmanek, 2011). The introduction of species has been carried for horticulture, forestry, food, and agroforestry (Richardson & Rejmanek, 2011). These deliberated introductions, besides being the main cause of species transportation outside their natural ranges (Pysek et al., 2008), are highly relevant, because as species of interest, resources for their successful establishment and propagation are ensured (Reichard & White, 2001), enhancing their naturalization. This interest is responsible for most problems surrounding tree invasions. Because of their introduction purposes, they are considered crops of high cultural, commercial, or aesthetic importance in those regions where introduction occurred, but in other regions, same species might be considered weeds, creating conflict of interest, which most of the time stagnate management efforts (Richardson et al., 2014). Some of the most relevant trees in terms of commercial use are listed as invaders in other regions. Fabaceae (*Acacia*, particularly) and Pinaceae (mainly *Pinus*) families are commonly listed as invasive trees globally. Haysom and Murphy (2003) stated that a considerable portion of species introduced with forestry purposes (total of 458 species) were already invading (61%) or naturalized (9%). South America arises as the third region with the highest number of introduced species with forestry purposes (180 approx.), after Asia (190) and Africa (219).

Although it is hard to predict the future spread of invasive tree species, there are tools available, which could allow the prediction of their spread in invaded regions. The magnitude of spatial spread (a proxy of invasiveness) can be predicted from species climatic requirement (climatic niche) and can be represented by species distribution models (SDMs). A species niche, as defined by Peterson et al. (2011), is defined as the ecological conditions that a species requires to maintain populations in a given region, together with the impacts that the species has on its resources, other interacting species, habitats, and environment. Species distribution models (SDMs) are one of the most popular tools to predict species potential distribution (Pearson & Dawson, 2003; Phillips, Anderson, & Shapire, 2006; Soberón & Nakamura, 2009). The conceptual basis of this approach is niche–biotope duality, that is, the possibility that niche requirements can be projected to the geographic space (Colwell & Rangel, 2009) and its aim is to predict environmental suitability in the geographic space (Peterson & Holt, 2003; Phillips et al., 2006). SDMs have been largely used in invasion ecology (Broennimann et al., 2007; Gallien, Münkemüller, Albert, Boulangeat, & Thuiller, 2010; Peterson, Papes, & Kluza, 2003; Reed, Meece, Archer, & Peterson, 2008; Thuiller et al., 2005). Two assumptions underlie these models: (a) Introduced species are in a biogeographic equilibrium with the new environment, which means that species are able to colonize every suitable habitat that exists in the new range and (b) niche conservatism, which means that we can transfer the model only to analogue climatic regions (Wiens et al., 2010).

One way to use SDM in invasion ecology is to project the native niche (niche of the species in its native range) in the invaded range (geographic area where the species is established and invading) and to compare it with the invaded niche (niche of the species in the invaded range) projected directly in the same invaded range (Broennimann et al., 2007; Fitzpatrick, Weltzin, Sanders, & Dunn, 2007). This approach has been profusely used to test niche conservatism, the tendency of species to maintain ancestral ecological requirements (Broennimann et al., 2012; Broennimann et al., 2007; Petitpierre et al., 2012). According to Petitpierre et al. (2012), overlapping the invaded and native niche in the environmental space allows the identification of three areas: (a) stability niche area (S): proportion shared between native and invaded niches, corresponding to the invaded niche that overlaps with the native niche (it is an estimation of niche conservatism), (b) unfilled niche area (U): fraction of the native niche, not shared with the invaded niche, and (c) the expansion area (E): fraction of the invaded niche not shared with the native niche. This area corresponds to the degree of niche shift, showing new environments occupied by the species in the invaded range. Using Broennimann et al. (2012) and Petitpierre et al. (2012) approaches combined allows to determine the invasive potential of a species in the invaded range. If the niche is conserved, then the introduced species will occupy only those geographic areas which share the climatic conditions of the native niche. If, on the other hand, the niche is not conserved, two situations can occur: one, where the invaded niche is contained by the native niche, which means that there is a unfilled area, available for colonization (the invasion is still an ongoing process) or second, where the invaded niche is partially or completely “outside” the native niche, meaning that the species is growing under new climatic conditions, different from those of the native niche. All these analyses are carried and interpreted in the environmental or niche space.

A second approach, based on species occurrences and the SDMs, compares the global niche model, developed considering the totality of presences known for the species worldwide (Gallien, Douzet, Pratte, Zimmermann, & Thullier, 2012), with the regional niche model, developed considering only the presences known for the species in the invaded range. Global niche models are a *proxy* of the fundamental niche, while the regional niche model is considered a proxy of the realized niche (Vetaas, 2002). Using a combination of both global and regional niche models (thus fundamental and realized niches) will improve the estimation of the potential distribution of a species in a given invaded range. The proposal of Gallien et al. (2012) allows the classification of the invasive stage of a species into four categories, and also projecting them in the geographic space (SDM): (a) When the presence of the species is predicted by both the global and the regional SDMs, it is considered to be at equilibrium with the new environment (it is predicted to be contained by the realized niche, inside the fundamental niche); (b) when the occurrence is not predicted by the global nor the regional SDM, the species is considered to be occupying unsuitable habitats; (c) when the occurrence of the species is predicted only by the global SDM, but not by the regional SDM, the species is considered to be in a colonizing stage, where not all available environments have been occupied yet; or (d) when the species occurrences are predicted by the regional SDM, but not by the global SDM, it is considered to be expanding to new environments, badly predicted by the global niche. This approach is also interesting because it recognizes the complex nature of invasive processes and consequently relaxes the assumptions of biogeographical equilibrium and niche conservatism.

*Acacia dealbata* Link (Fabaceae: Mimosoideae) is a tree, native to the southeast coast of Australia (Victoria, New South Wales, and Tasmania) (Boland et al., 2015); it has been considered one of the most invasive species of the genera. Its presence has been registered in at least 34 countries in all the Continents; it has already been classified as invasive in Turkey, India, Sri Lanka, South Africa, Madagascar, United States, Argentina, Chile, Bolivia, Uruguay, Brasil, France, Italy, Spain, Portugal, and New Zealand (CABI, 2018; Herrera, Goncalves, Pauchard, & Bustamante, 2016). It is a prolific species (sprouts and reproduces sexually), forming a permanent seed bank, which is readily available after fire or any significant perturbation. Seed is dispersed by water, animals, and human activities, and it also coppices easily (Fuentes et al., 2014). It invades mostly grasslands, riparian habitats, open forests, and disturbed sites (Weber, 2017), forming dense thickets, disrupting water flow, suppressing native vegetation, and increasing soil erosion along stream banks (Fuentes‐Ramirez, Pauchard, Cavieres, & García, 2011). In Chile, *Acacia dealbata* was introduced around 1869 (Fuentes et al., 2014), for ornamental purposes. Other uses include soil improvement, dunes stabilization, and erosion control (Jaksic & Castro, 2014). In Chile, its presence has been registered between Valparaiso and Los Lagos regions (32°00′00″S and 44°00′00″S), including Juan Fernandez island and Easter Island; in Mediterranean ranges, it is associated mainly with riparian habitats, roadsides, and anthropogenic disturbances (Matthei, 1995; Pauchard & Maheu‐Giroux, 2007; Peña, Langdon, & Pauchard, 2007).

In Chile, although studies about *A. dealbata* have addressed its invasion patterns and potential impacts (Fuentes‐Ramirez et al., 2011; Fuentes‐Ramírez, Pauchard, Marticorena, & Sanchez, 2010; Pauchard & Maheu‐Giroux, 2007; Peña et al., 2007), there is no information regarding its biogeography and the climatic requirements that best explain its distribution. We want to know whether the invasion process of *A. dealbata* in south‐central Chile has already reached an equilibrium, whether its present distribution will not change greatly in the future, or whether the species is still spreading and will increase its distribution range in Chile. The aim of this study was to examine the climatic niche of *Acacia dealbata*, (a) in the environmental space, testing for niche conservatism, using the Broennimann et al. (2012) and Petitpierre et al. (2012) approaches of native‐invaded niche contrasts, which will allow us to determine whether the species is establishing in south‐central Chile under the same climatic conditions it occupies in the native range, and (b) in the geographic space, testing for geographic equilibrium and the invasion stages of the species in south‐central Chile, using Gallien et al. (2012) approach to compare the global and regional (invaded) SDM. We also aim to examine potential constraints to *A. dealbata* distribution in its native range using the global‐native range niche contrast, which could explain the distribution of the species in south‐central Chile.

## 2 METHODS

### 2.1 Study area

The study was conducted in south‐central Chile, between the O'Higgins (34°0″0′S) and the Aysén (47°0″0′S) regions (290,519 km^2^). This includes the largest forestry plantations surface in Chile (2,312,696 ha), accounting for 94.5% of all forestry plantations surface (INFOR, 2014). This geographic range encompasses a wide climatic differentiation, ranging from a warm temperate climate with a dry season and great cloudiness in the north, a rainy temperate climate in the central part, and a cold steppe in the southern extreme of the study area (DGAC, 2006). In terms of vegetation, Luebert and Pliscoff (2006) describe five vegetational formations in the study area: sclerophyllous forests, deciduous forests, broad‐leaved forests, evergreen forests, and the steppe. Also, 13,920,280 ha of native forests (CONAF, 2017) are present in the study area and concentrated in the Los Lagos and Aysén regions, with more than 50% of forests surface of Chile.

### 2.2 Data collection

#### 2.2.1 Species occurrences

Data on the presence of *Acacia dealbata* at a global scale were recorded from different sources during April 2016:

- Global Biodiversity Information Facility (www.gibf.org)
- DC Herbarium at the Smithsonian Institution (http://botany.si.edu/dcflora/dcherbarium.htm)
- Consortium of California Herbaria (http://ucjeps.berkeley.edu/consortium/)
- Australia Virtual Herbarium (http://avh.chah.org.au/)
- University of Washington Herbarium at the Burke Museum (http://www.burkemuseum.org/herbarium)
- the Integrated Digitized Biocollections (https://www.idigbio.org/)
- SpeciesLink (www.splink.org.br)
- Tropicos (www.tropicos.org)
- Intermountain Region Herbarium Network (www.intermountainbiota.org)
- The New York Botanic Garden Virtual Herbarium (https://www.nybg.org/)
- Consortium of Pacific Northwest Herbarium (www.pnwherbaria.org)

Data were carefully filtered according to the following criteria: (a) The data contained associated georeferenced information (e.g., datum), (b) they were recorded after 1950 to minimize erroneous georeferenced information, and (c) there is an associated voucher or were labeled under the name of the botanist who determined the sample. All duplicated records were also eliminated, and a subset of the occurrence data was created, based on the Euclidean distance between points, in order to consider just one point per cell during the modeling process. All points within 9 km of distance were removed from the data, resulting in a resolution of 2.5 arc minutes (about 4.5 km). These analyses were carried out using R software (version 3.3.1) (R Core Team, 2016).

For the native range, occurrence data were selected from the global data set. The native range was identified from the literature (Boland et al., 2015), and then, all occurrence points within that defined area were selected and extracted using ArcGis 10.2. A polygon was then created using the minimum bounding geometry tool with the convex hull geometry type. This polygon was then used as a mask to extract the native range from all the environmental layers.

For the study area (south‐central Chile), online occurrence data were complemented with field surveys, distinguishing planted from naturalized individuals, and excluding all urban areas, and gardens or ornamental individuals in rural areas (Ramirez‐Albores, Bustamante, & Badano, 2016). A total of 300 sampling points were randomly distributed through the study area using ArcGis (v. 10.2), and then, the total number was reduced according to its accessibility (no more than 1 km between each point and a primary, secondary, or tertiary road), resulting in a sample of 81 sampling points to visit. Field surveys were carried out between October and December 2017. At each sampling point, we looked for *A. dealbata* individuals within our visual ranges and georeferenced the site where the species was recorded. Occurrences along the roads were registered as well, giving a total (sampling point plus side of the roads) 551 visiting points (120 and 431 absences). Only a small fraction of the presences registered (6 of 120) corresponded to planted individuals. Considering that this differentiation between planted and naturalized individuals is available for data collected in the field, but not for global and native occurrences gathered online, we decided to include and treat all data without any differentiation. It is known, and it will be considered during result interpretation that the result could be affected by this decision, overestimating the potential distribution of the species to areas where it could not survive without human assistance.

After gathering all data available (4,617 global occurrences and 120 regional occurrences), we eliminated duplicated and erroneous data (it falls in the ocean, for example), applied described filters, and reduced the data to the determined resolution (2.5 arc minutes). Finally, analyses were performed based on 1,384 global occurrences of *A. dealbata*, with a fraction of 698 of them being native and 97 regional occurrences in the invaded range (south‐central Chile).

#### 2.2.2 Environmental layers

Bioclimatic variables were obtained from the WorldClim database (www.worldclim.org/, public repository online) with a spatial resolution of 2.5 arc minutes (Hijmans, Cameron, Parra, Jones, & Jarvis, 2005). The data set included 19 bioclimatic variables summarizing temperature and precipitation data. Since variable collinearity may lead to overfitting, we checked for cross‐correlation between all possible pairs of variables using the Pearson correlation test using ENMTools software, version 1.44 (Warren, Glor, & Turelli, 2008). Only one variable from highly correlated pairs of variables (*r*. > 0.70) was included in the model, allowing us to minimize redundancy (Warren et al., 2008). We finally selected seven variables, based on the correlation analyses mentioned above and their relevance for tree ecology: annual mean temperature (BIO1), maximum temperature of the warmest month (BIO5), minimum temperature of the coldest month (BIO6), annual precipitation (BIO12), precipitation of the wettest month (BIO13), precipitation of the driest month (BIO14), and precipitation seasonality (BIO15).

### 2.3 Niche conservatism

Following Broennimann et al. (2012), a principal components analysis (PCA), calibrated on the entire environmental space of both native and invaded areas (Chile) (PCA‐env), was used to determine whether *A. dealbata* native niche is conserved in the invaded range (Chile). Two sets of occurrence points were prepared, one for Australia (native range) and other for south‐central Chile (invaded range). The environmental space was divided into 100 × 100 cells, and all occurrence points were converted into density values, using a kernel function to smooth the distribution of the densities. Then, 10,000 random points (i.e., pseudo‐absences) were generated, to estimate the density of available environments in the environmental space. Based on the values of occurrence and available environments densities, an occupancy index was estimated. This occupancy index was plotted on the environmental space, for both the native and the invaded ranges. Niche overlap (shared areas between two niches) between invaded and native niches was assessed using three approaches:

1. Schoener's D overlap index, ranging from 0 (no overlap) to 1 (complete overlap),
2. Niche equivalency, which determines whether niches of two entities in two geographical ranges are equivalent or whether the niche overlap is constant when randomly reallocating the occurrences of both entities among the two ranges. A significant value means a rejection of the hypothesis that the two niches are identical (Broennimann et al., 2012; Warren et al., 2008).
3. Niche similarity, which asks whether the environmental niche models generated from two populations are identical or merely more similar than expected by chance (Warren, Glor, & Turelli, 2010). A statistically significant comparison in both directions (native to invaded and invaded to native) allows to consider that both niches are more similar than expected by chance.

Complementary, according to Petitpierre et al. (2012), overlapping the invaded and native niche in the environmental space allowed us to identify three areas: (a) stability niche area (S), (b) unfilled niche area (U), and (c) the expansion area (E). Petitpierre et al.'s (2012) framework was limited to analogous climates between the native and invaded ranges, but following Webber, Maitre, and Kriticos (2012), we decided to consider all available climates in both regions, including nonanalogous climates. All niche analyses were carried out using R software (version 3.3.1) (R Core Team, 2016), with the BIOMOD, ade4, adehabitat, sp, gam, MASS, mvtnorm, gbm, and dismo packages.

We also estimated niche similarity (Warren et al., 2008) between the environmental space of the 120 occurrences versus the environmental space of the 431 absences registered in the invaded range (study area). If both spaces are more similar than expected by chance, then the species is not in equilibrium and more areas remain to be invaded; if similarity is not significantly different by chance, then the current presence of the species is in biogeographical equilibrium.

### 2.4 Climatic analogy

The climatic analogy between the native and invaded regions was evaluated to detect the existence of novel climates in the invaded range. This analysis gives us the chance of mapping in the geographic space, the results of the comparison of climatic spaces (Elith, Kearney, & Phillips, 2010). We used the multivariate environmental similarity surface (MESS) analysis, integrated in Maxent (Phillips et al., 2006), which represents how similar a point is to a reference set of points, with respect to a set of predictor variables. It allows negative values—which will show sites where at least one variable has a value that is outside the range of environments over the reference set, so these are novel environments. The values in the MESS are influenced by the full distribution of the reference points, so that sites within the environmental range of the reference points but in relatively unusual environments will have a smaller value than those in very common environments (Elith et al., 2010). Two results will be obtained: In one map (MESS), areas shown in red present one or more variables outside the range present in the training data (native range). The second map shows the most dissimilar variable (MoD), that is, the one that is furthest outside its training range (Elith et al., 2010). Values of the variables for both nonanalogous regions were compared using a Student *t* test, following Goncalves et al. (2014).

### 2.5 Species distribution models (SDMs)

We used SDM to predict potential suitable areas for *A*.* dealbata* in Chile predicted from its native range, from the invaded range (Chile) and for the global range. We used Maxent (Phillips et al., 2006), a machine learning software that assesses the distribution probability of a species, by estimating the distribution probability of maximal entropy. This software has been proven to perform better than other software commonly used with only presences data sets (Elith et al., 2006; Graham et al., 2008; Ortega‐Huerta & Peterson, 2008). Model accuracy was tested using a cross‐validation method. Occurrence data for each region were divided into two parts: 75% for training and 25% for testing the model. Then, model performance was tested using the AUC (area under the ROC curve), ranging from 0.5 for a model that performs no better than chance to 1, a model with a perfect ability to predict the species presence (Evans, Smith, Flynn, & Donoghue, 2009; Phillips et al., 2006). For SDM regularization, we smoothed the models to avoid overparameterization (Elith et al., 2011; Phillips et al., 2006). A threshold was established, defined by the 10% percentile of probability of occurrence (Peterson et al., 2011). All values, below that threshold, were discarded under the assumption that these figures represented unsuitable climatic zones. Each model was the average of 50 replicates. Three SDMs were constructed: global, regional, and native SDMs. The global SDM was constructed using all global occurrences and a global background (the complete environmental layers obtained from WorldClim). The regional SDM considered occurrences in south‐central Chile (the invaded niche) with the study area background. The native SDM considered native occurrences and the native background. The native‐invaded range contrast was used to put into geographic space the niche conservatism analyses; the global‐invaded range contrast was used to test invasive stages of the species in south‐central Chile. The global‐native range contrast was used to test for constraints of species distribution in its native range.

### 2.6 Invasion stage

Global and regional niches were compared following Gallien et al. (2012), thus allowing us to infer the stage of invasion in the niche space. To do that, probability occurrences predicted from both models were correlated on real occurrence points registered in the invaded range. We also mapped the zones registered as equilibrium, colonization, adaptation, and sink populations.

## 3 RESULTS

### 3.1 Niche conservatism

**Figure 1:**
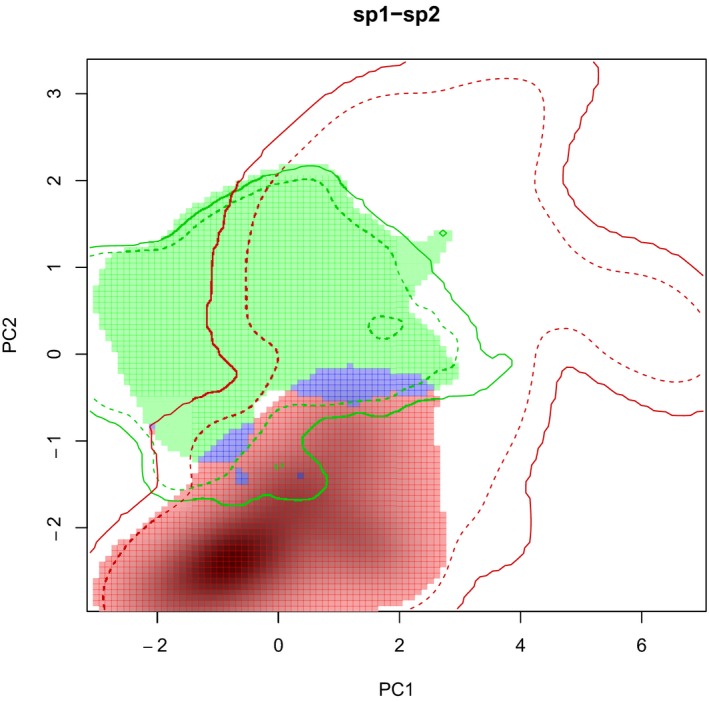
PCA‐env results showing the climatic niche of *A. dealbata* in its native range (in green), in the invaded range (in red), and the rather small overlapping area of both niches (in blue). Solid and dashed lines show 100% and 50% of the climatic envelope of each region. According to Petitpierre et al.'s (2012) framework, the green area corresponds to the unfilled niche (U), the red area to the expanded niche (E), and the blue area to the stability area (S)

**Figure 2:**
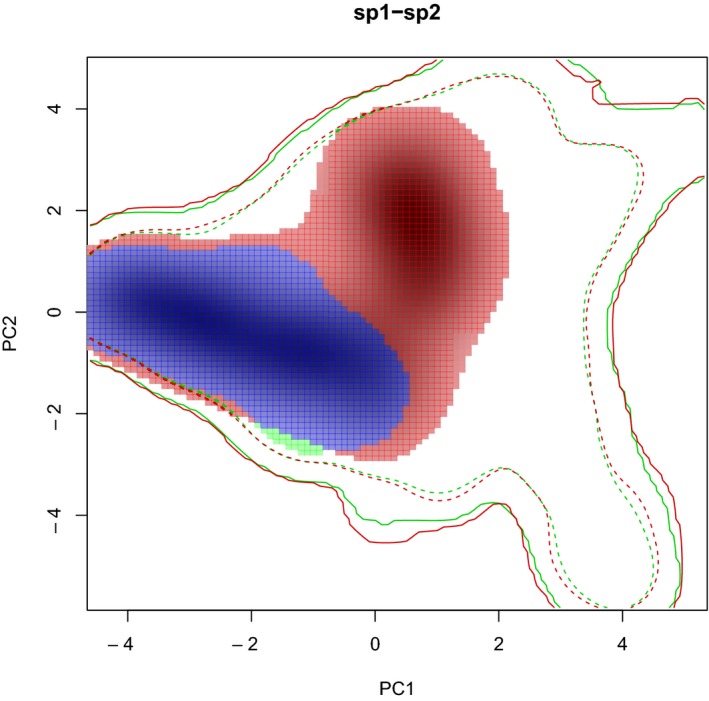
PCA‐env results showing the environmental space of the 120 occurrences (in green) and 431 absences (in red) of *A. dealbata* in the invaded range. The overlapping area of both niches is shown in blue. Solid and dashed lines show 100% and 50% of the climatic envelope of each region

The PCA‐env showed that axes, PC1 and PC2, explained more than 75% of the total variance. BIO15 (precipitation seasonality) explained PC2, and BIO1 (annual mean temperature), BIO5 (maximum temperature of the warmest month), and BIO12 (annual precipitation) explained PC1. Native and invaded range niches were quite different. Schoener's D overlap index = 0.08, meaning almost no overlap between the two compared niches. The equivalency test (*p* = 0.02) resulted in a rejection of the equivalency hypothesis. The similarity test (*p* = 0.396 (niche similarity of Australia to Chile); *p* = 0.337 (niche similarity of Chile to Australia)) showed the same trend. Results were not statistically significant, which means that both niches are not more similar than expected by chance. These niche differences are better presented analyzing a low fraction of niche overlap (stability 0.024), the high fraction exclusive to invaded niche (expansion 0.976) and the high fraction exclusive to native niche (unfilling 0.992) (Figure 1). The environment of the occurrences and the environment of the absences resulted quite similar (*D* = 0.55, *p* = 0.02 of presences to absences, *p* = 0.02 of absences to presences)) (Figure 2).

### 3.2 Climatic analogy

**Figure 3:**
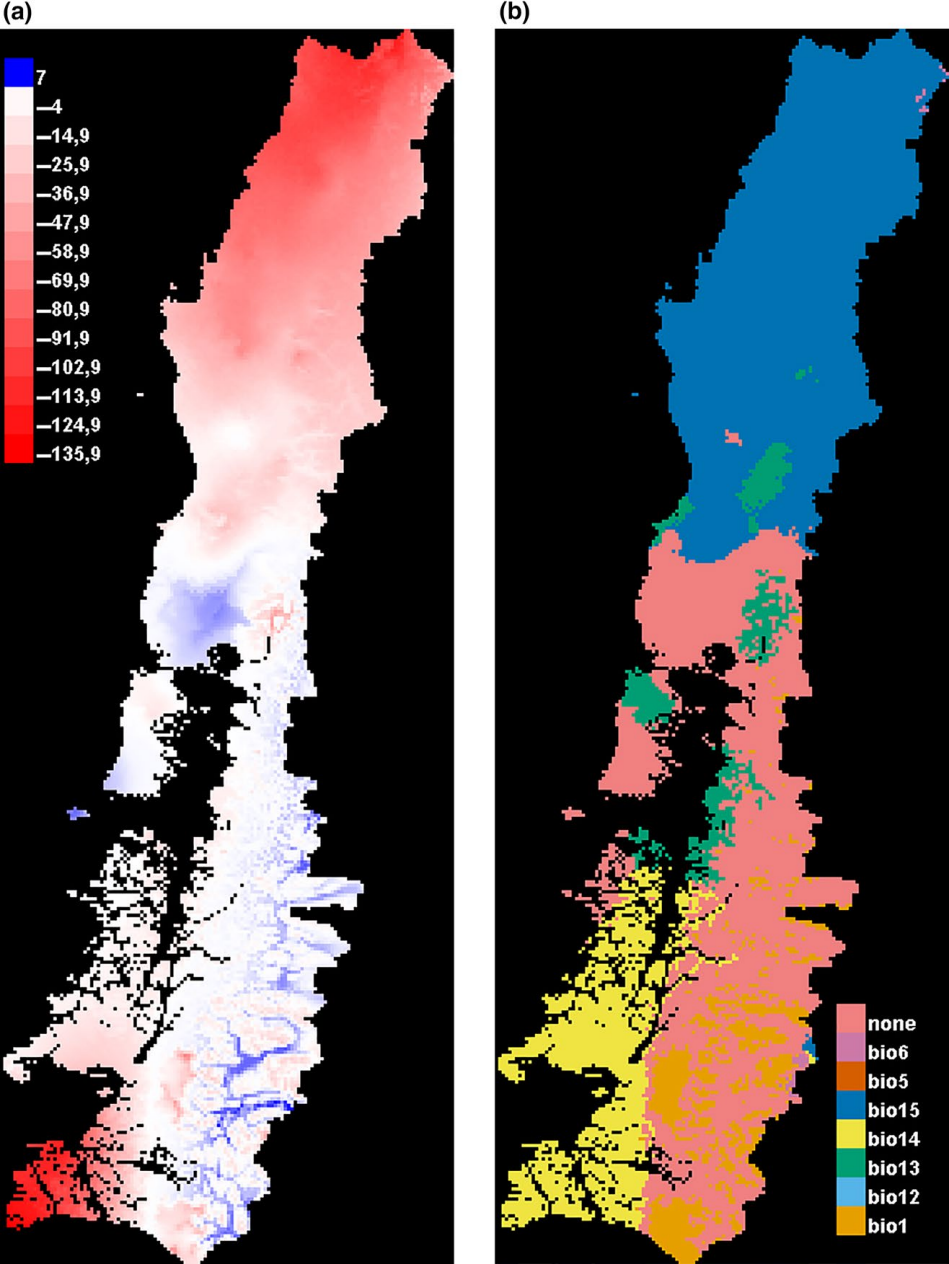
Results of the (a) MESS analysis showing in red areas, the different levels of dissimilarity when outside the range of the reference points, for us, the native range, that is, red areas present climatic variable(s) whose values are different from those present in the native range for the same climatic variable, and (b) MoD shows the most dissimilar variable, the one that is furthest outside its training range, that is, shows the climatic variable which present the higher differences between the native and invaded ranges (Elith et al., 2010) In our case, the main climatic difference between the native and invaded ranges locates in the north area of the study area, and it is given by BIO15 (precipitation seasonality) shown in blue

According to the MESS analysis, there are two areas in the invaded region that present nonanalogous conditions to those present in the native range. The main one locates in the northern central Chile and a smaller part in the most southern range (see zones in red) (Figure 3a). BIO 15 (precipitation seasonality) and BIO 14 (precipitation of the driest month) are the two variables which present the most dissimilar values related to those areas (Figure 3b). BIO14 was discarded for further analyses, because no occurrences were registered in the affected area. The Student *t* test was performed using BIO 15 (precipitation seasonality) data values extracted for each occurrence point, in both the invaded and native ranges. Results showed a mean of 22.4% of precipitation seasonality in the native range versus 78.64% of precipitation seasonality in the invaded range (*p* < 0.01). Interestingly, 65% of the observed occurrences registered in the invaded area were located in nonanalogous climate region.

### 3.3 SDM contrasts

**Figure 4:**
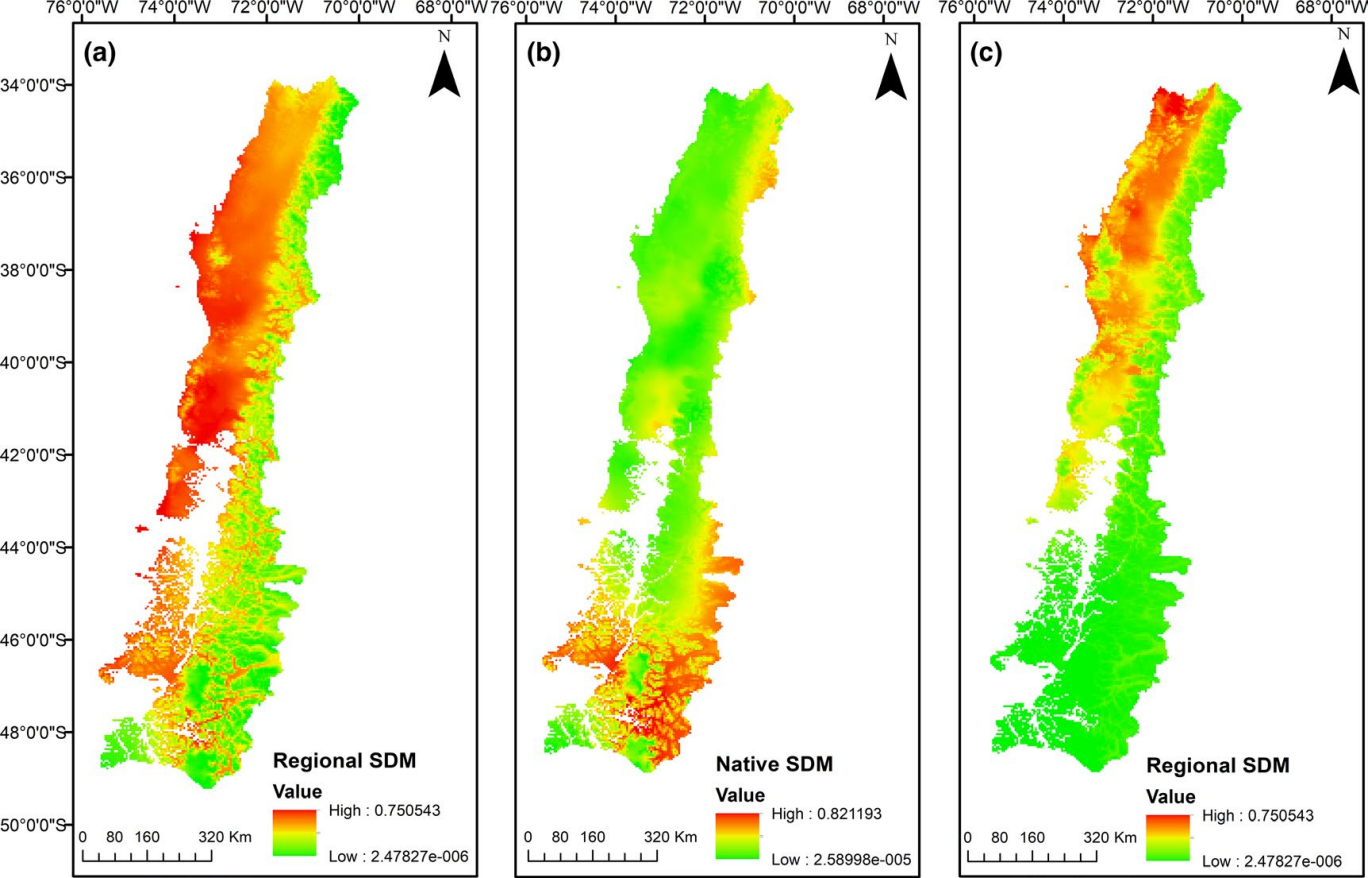
Species distribution models (SDM) for *A. dealbata* based on (a) global occurrences with a global background, (b) native occurrences with a native background, and (c) regional occurrences (invaded region) with a regional background. Red areas represent higher and green areas represent lower occurrence probabilities. Both (a) and (b) represent the predicted future potential distribution of *A. dealbata* in the study area, based on its global or native presences, while (c), on the other hand, is a representation of the current status of *A. dealbata* in south‐central Chile

**Figure 5:**
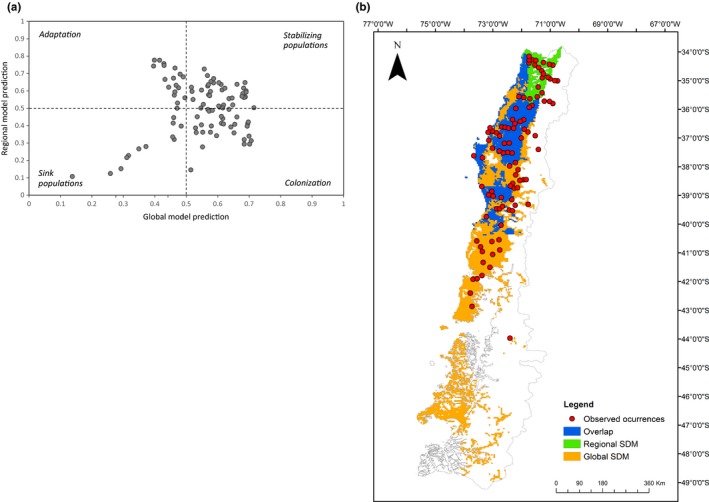
Invasion stages for *A. dealbata* in the invaded region. (a) shows plotted values of global and regional models’ predictions for each one of the observed occurrences (red dots), (b) shows the geographical representation of the global niche in green, regional niche in orange and areas predicted by both global and regional models (overlap area) in blue. Gray areas represent areas outside of both niches. Following Gallien et al. (2012), both SDMs were mapped considering an occurrence probability threshold of 0.5

The global, invaded, and native SDMs showed a very good fit (AUC: 0.947, 0.835, and 0.807, respectively). As Table 1 shows, variable contribution changed between the three models. BIO 6 (minimum temperature of the coldest month) presents the higher contribution to the global SDM. For the invaded SDM (in Chile) and the native SDM, on the other hand, the higher contributions were related to BIO1 (annual mean temperature). Particularly in south‐central Chile, the global SDM (see Appendix S1) presented a suitable area of 175,957 km^2^ (threshold of 0.3, Figure 4a) (see Appendix S2), and the native SDM projected a suitable area of 118,900 km^2 ^(threshold of 0.2; Figure 4b). The invaded SDM projected a suitable area of 104,340 km^2 ^(threshold of 0.3; Figure 5c) (see Appendix S3). It is clear that there existed a spatial discordance between SDMs projected in Chile. First, the native SDM projected the highest occurrence probabilities between 44°S and approx. 49°S latitude. Second, in the invaded range SDMs, higher probabilities occur between 34°S and 40°S. Third, the global SDM projected higher occurrence probabilities between at least 34°S and 49°S latitude (see Appendix S4).

**Table 1:** Relative contribution of each environmental variable to the Maxent model

Variable	Contribution (%)		
	Global SDM	Native SDM	Invaded SDM
BIO1	14	51.2	48.3
BIO5	2.3	7.6	9.6
BIO6	77.3	26.1	7.6
BIO12	1.6	3.2	2.7
BIO13	0.5	4.9	3.4
BIO14	1.3	5.9	8.8
BIO15	2.9	1.1	19.6

Note

Values shown are averages over replicate runs.

### 3.4 Invasion stage

**Figure 6:**
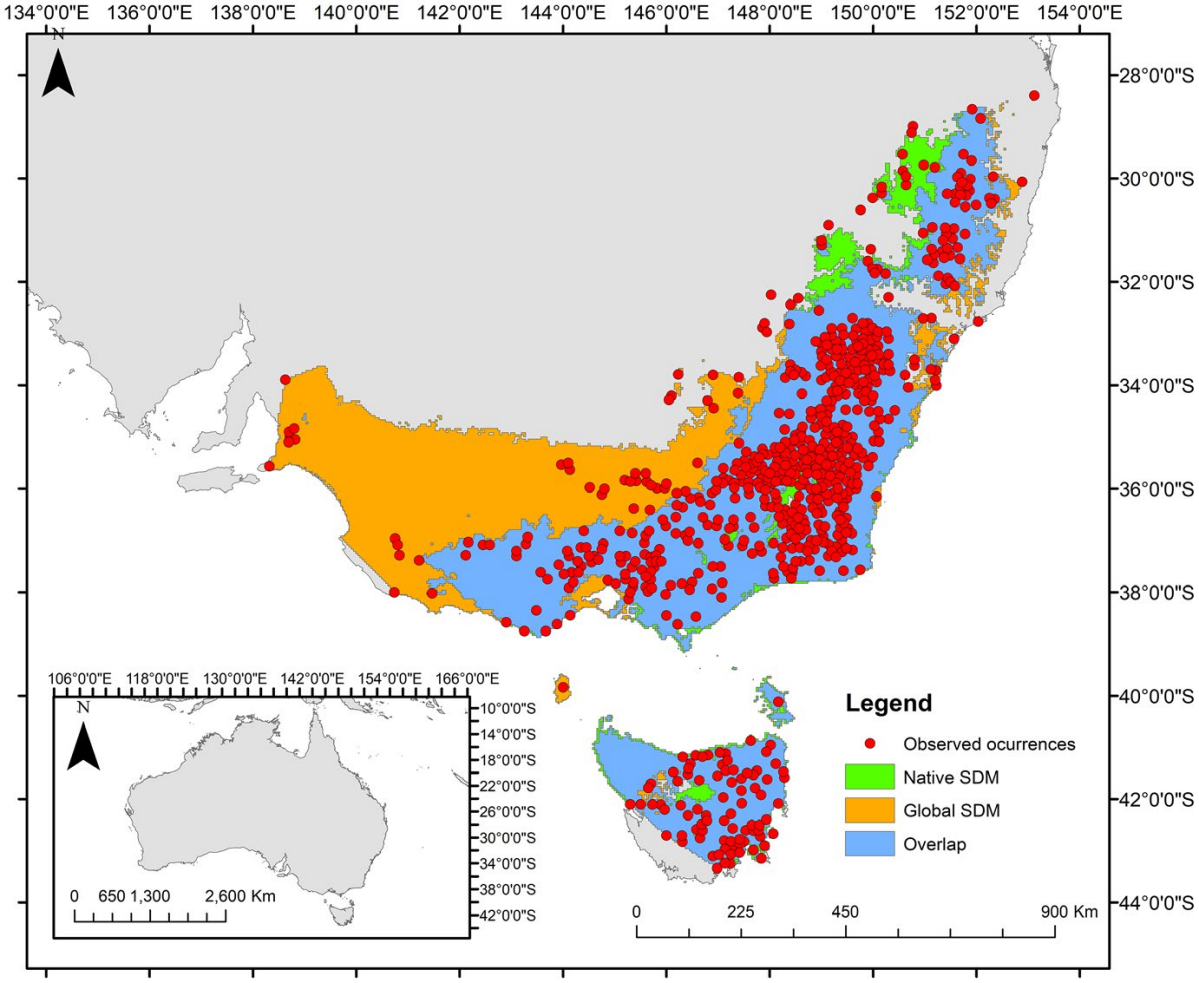
Potential distribution of *A. dealbata* in its native range based on global occurrences (in orange), native occurrences (green areas), and area predicted by both global and native SDMs (overlap area) in blue. Red dots represent native occurrences

Following Gallien et al. (2012), we observed that 30.9% of occurrences are in colonization stage, 38.1% are in stabilization zone, 18.6% represent local adaptations, and 12.4% correspond to sink populations (Figure 5a). The geographical projection of these zones depicted approximately the same pattern (Figure 5b). The global SDM projected a potential area of 562,991 km^2^ in the native range, while the native SDM only projected an area of 397,171 km^2^. The overlapping area of both SDMs covers 360,826 km^2^. The global niche model predicted an area of 30% higher than that predicted by the native SDM (Figure 6).

## 4 DISCUSSION

Niche requirements, climatic suitability, and geographic distribution are critical to understand *A. dealbata* invasion process in south‐central Chile. We assessed the current invasive status of this species in south‐central area of Chile using three niche contrasts: native‐invaded, global‐invaded, and global‐native ranges. Next, we will discuss the results of each contrast as well as the implications for the invasive potential of this exotic tree.

Our results showed that the species has shifted its climatic niche in south‐central Chile, its current distribution is not in a biogeographic equilibrium; therefore, there are more suitable habitats to be colonized in the future. If niche shifts in the invaded range, then estimations of potential distributions based solely on the native range are not amenable to be used (Petitpierre et al., 2012). This prediction resulted in a subestimation of 32% of the potential distribution, when compared with the global SDM. Moreover, we detected spatial discordances using native‐invaded niche range contrast: While native niche projected distribution to the south Patagonian regions, the invaded niche projected distribution to Mediterranean zones of central Chile. Therefore, in south‐central Chile, there are conditions still available for *A. dealbata* to establish. Only a 0.24% (Stability zone) of the niche is at equilibrium (conditions are shared in both the native and the invaded niches), which represents 9,504 km^2^ located in the Los Lagos region. There are still more than 100,000 km^2^ (99.2%) of unfilling zone south of the Los Lagos region (Chilean Patagonia) and 94,836 km^2 ^(97.6%) of Mediterranean climate at the northern areas of the study area (expansion zone), which have not been yet occupied by the species. The nonequilibrium conditions are reinforced by the climatic similarity between the points of presences (occurrences) and absences. The high proportion of unfilling zone might respond to dispersal limitation or the reduction of human disturbance to the northern zone (INE, 2018). In fact, *Acacia dealbata* invasion has already been associated with human activities, such as forestry and road presences (Pauchard & Maheu‐Giroux, 2007). Including such variables in future analyses could explain low colonization into southern areas. The surprisingly large expansion to the north area, on the other side, which could be associated to higher human density (INE, 2018) among other factors, seems to be highly correlated to climate factors. Including the human footprint variable would be a relevant factor to improve the models presented here, revealing the mechanisms which could be acting behind the expansion and colonization zones of the species distribution. Another factor which could be acting behind niche differences is the fact that *A. dealbata* has two subspecies in its native range, *A. dealbata* subsp. *dealbata* and *A. dealbata* subsp. *subalina*. A difference in the proportions of both subspecies in the occurrence points used to model the niches and potential distributions could explain the resulting different niches and potential ranges. Unfortunately, it is not easy to clear this matter, since most of occurrence data available online do not include the information at the subspecies level.

According to our results on climate analogy, the main driver of the niche shift appears to be precipitation seasonality, leading it toward more variable precipitation. Then, areas with marked dry and wet seasons could be more suitable for *A. dealbata* establishment in Chile, under conditions not present in the native range. Interestingly, the global‐native SDM contrast showed that only 9% of the native climatic conditions are not represented in its global distribution. But on the other side, its native SDM projects an area 30% smaller than the global SDM. These could explain the species expansion zones (containing 65% of occurrences), which could be the result of a reduced native range and thus climatic conditions not included in the native climatic niche. We sustain that better predictions of invasion in Chile can be made using global niche model, including occurrences of Chile; in that way, we use the totality of environments that the species has colonized worldwide.

The lower representation of suitable environments in the native range relative to global distribution opens interesting issues about the ecology and evolution of *A. dealbata*. It is possible that this species is limited by dispersal or negative biotic interactions (Le Maitre, Thullier, & Schonegevel, 2008; Peterson et al., 2011) or because the climate conditions that species can colonize in other regions of the world existed in the past and no longer exist today. This last fact that has been well documented in other trees species (Svenning & Skov, 2004) can help us to anticipate potential invaders, examining their potentiality to survive and reproduce in conditions other than those they find in their native range.

The stage of the invasion process also indicates that the *A. dealbata* in south‐central Chile is clearly far from stabilizing. Around 38% of occurrences are stable populations, another 30% correspond to colonizing populations, 18% correspond to local adaptations, and 12% are sink populations. The overlapping zone of both SDMs, where populations are stable, corresponds to the Mediterranean areas of Chile, local adaptations are occurring to the northern study area, and colonization populations are those located to the south portion of the study area. These results corroborate our result of the native‐invaded range niche contrast.

Species distribution models have once more proven to be a useful tool when planning conservation actions. Recently, a study carried by Fernandes et al. (2019) showed the significance of using SDM in a transfrontier context to anticipate invasions, particularly of *A. dealbata* in Portugal and Spain. Wilson et al. (2011) recommend that *A. dealbata* (with an extremely high invasive potential) should be identified and removed of all climatically suitable countries where they are not yet widespread. The same advice should be considered at smaller scales, considering suitable regions or areas, regardless of administrative borders. We then highly recommend that management alternatives, at the regional scale, should focus on prevention, avoiding, for example, the introduction of *A. dealbata* to the south of the Los Lagos region, where the species has not spread yet. Early detection will be also relevant at smaller scales, near biodiversity conservation sites (Kull, Tassin, Rambeloarisoa, & Sarrailh, 2008) or plantations edges, where owners should execute monitoring or management activities. Potential distribution based on climatic suitability will be fundamental to identify those areas in south‐central Chile which should be permanently monitored for signs of recruitment. More detailed studies, at a local scale, should be carried to determine factors acting behind the invasion process. Microclimatic conditions, biotic interactions, or human activities should be incorporated to elucidate the best control efforts at a local scale. The whole introduced range of *A. dealbata* in south‐central Chile should also be considered for further analyses, because the choice of the extension studied will strongly condition the niche–biotope duality, and then the available environments, and potential niche changes (Guisan, Petitpierre, Broennimann, Daehler, & Kyeffer, 2014).

*Acacia dealbata* climatic niche in south‐central Chile has shifted, its current distribution is not in a biogeographic equilibrium; therefore, there are more suitable habitats to be colonized in the future. The invasion process is far from stabilizing. There are stable and still colonizing populations, but others are adapting to the new environmental conditions. We recommend that management options should focus on prevention, avoiding, for example, the introduction of *A. dealbata* to Patagonia, where the species has not spread yet.

## CONFLICT OF INTERESTS

None declared.

## AUTHORS CONTRIBUTION

Bárbara Langdon and Ramiro Bustamante contributed to the idea and aim of this research. BL was responsible for the fieldwork and data collection, and analyses and preparation of the manuscript. RB and Aníbal Pauchard both collaborated with the analyses, the discussion of the results, and final comments to the document.

## Data Availability

All data related to this study are included or cited in the manuscript. Environmental layers are available online at the WorldClim database, and occurrence records are available at different online sources cited in the main text (represented in Figure S1 from Appendix S4). Raster grids with the species distribution models (SDMs) are available as Supporting information (Appendices S1–S3).
