# Supplementary material for: Acacia dealbata invasion in Chile: Surprises from climatic niche and species distribution models
Source: Ecol Evol. 2019 Jun 23;9(13):7562–73. doi: 10.1002/ece3.5295 (PMC6635919; doi:10.1002/ece3.5295)
Supplement: Supplementary file 4 [file ECE3-9-7562-s004.docx]

**APPENDIX S1**


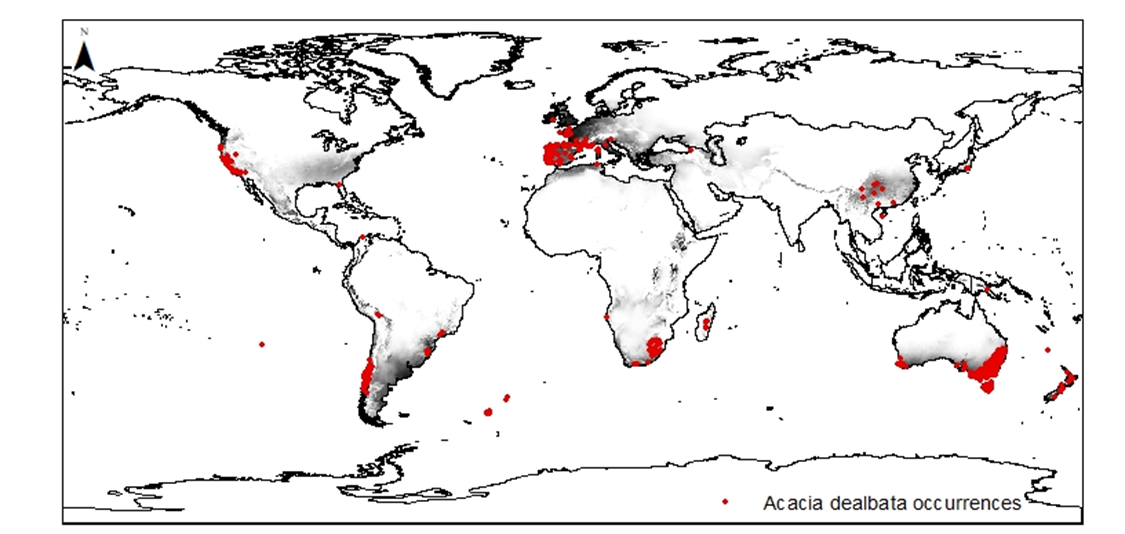


Figure S1.1 Global Spatial Distribution Model (SDM) of *Acacia dealbata*. Grey areas represent occurrence probabilities, with darker areas representing higher probabilities. Red dots represent *A. dealbata* occurrences.
